# The ChEMBL database as linked open data

**DOI:** 10.1186/1758-2946-5-23

**Published:** 2013-05-08

**Authors:** Egon L Willighagen, Andra Waagmeester, Ola Spjuth, Peter Ansell, Antony J Williams, Valery Tkachenko, Janna Hastings, Bin Chen, David J Wild

**Affiliations:** 1 Department of Bioinformatics - BiGCaT, Maastricht University, P.O. Box 616, UNS50 Box 19, NL-6200 MD, Maastricht, The Netherlands; 2 Department of Pharmaceutical Biosciences, Uppsala University, PO Box 591, SE-751 24, Uppsala, Sweden; 3 School of Information Technology and Electronic Engineering, University of Queensland, St Lucia, Qld 4072, Australia; 4 Royal Society of Chemistry, 904 Tamaras Circle, Wake Forest, NC 27587, USA; 5 Cheminformatics and Metabolism, European Bioinformatics Institute, Wellcome Trust Genome Campus, Hinxton, Cambridgeshire, CB10 1SD, UK; 6 School of Informatics and Computing, Indiana University, Bloomington, IN, USA

**Keywords:** ChEMBL, Bioactivity, Semantic web, Resource Description Framework, Linked Data

## Abstract

**Background:**

Making data available as Linked Data using Resource Description Framework (RDF) promotes integration with other web resources. RDF documents can natively link to related data, and others can link back using Uniform Resource Identifiers (URIs). RDF makes the data machine-readable and uses extensible vocabularies for additional information, making it easier to scale up inference and data analysis.

**Results:**

This paper describes recent developments in an ongoing project converting data from the ChEMBL database into RDF triples. Relative to earlier versions, this updated version of ChEMBL-RDF uses recently introduced ontologies, including CHEMINF and CiTO; exposes more information from the database; and is now available as dereferencable, linked data. To demonstrate these new features, we present novel use cases showing further integration with other web resources, including Bio2RDF, Chem2Bio2RDF, and ChemSpider, and showing the use of standard ontologies for querying.

**Conclusions:**

We have illustrated the advantages of using open standards and ontologies to link the ChEMBL database to other databases. Using those links and the knowledge encoded in standards and ontologies, the ChEMBL-RDF resource creates a foundation for integrated semantic web cheminformatics applications, such as the presented decision support.

## Background

The current scientific data deluge, in which datasets grow faster in size and complexity than scientists can keep up with, defines several new challenges for information systems. Scientists wish to discover new, unique, and significant patterns in datasets that explain biological phenomena not yet understood. The discovery of new patterns showing the causes and effects of various biological phenomena is often beyond the scope of single datasets. For example, systems biologists integrate micro-array differential expression datasets to biological pathways, using various other datasets to provide evidence for the links [1]. Another prominent example is drug discovery, in which a new unique chemical entity is designed or discovered based on a description of its required biological properties. This process requires the effective linkage of many scientific datasets that are both sparse and are growing independent of each other [2-4].

ChEMBL contains descriptions for biological activities involving over a million chemical entities, extracted primarily from scientific literature. This provides a unique resource for drug researchers [5,6]. It is updated on a fairly frequent basis as the existing data is further curated and new data is added. The ChEMBL dataset is available for download and can be browsed using a web interface and web services. The former requires scientists to import the data into a relational database, while the latter limits machine access to the data. ChEMBL also provides web services for programmatic access, but these are limited to the specific types of queries which are encoded in the web service API.

Using the right to download, modify, and redistribute the ChEMBL data, two independent teams have previously mapped the ChEMBL relational database to the Resource Description Framework (RDF), resulting in Chem2Bio2RDF [7] and ChEMBL-RDF [8]. RDF is a framework where knowledge is represented by small so-called triples, reflecting “facts”. A fact can be given on any topic, and RDF therefore talks about *things* very generically [9]. Things in RDF are represented by resources named with Uniform Resource Identifiers (URI). When the URI can be resolved, for example using the HTTP protocol, it creates *linked data*[10]. RDF is developed as an open standard by the World Wide Web Consortium, and provides a knowledge representation framework that applies to many, if not all, domains, and has been used successfully to integrate data for the life sciences.

This paper presents an update on the ChEMBL-RDF data set, including details of the latest structures and ontologies used to map ChEMBL to RDF, along with new use cases, showing links to other datasets using their RDF linked data URIs to further support research in the life sciences.

## Methods

The ChEMBL version used in this paper is ChEMBL 13, which was released on 29 February 2012. The SQL data dump provided by ChEMBL was inserted into a local MySQL database. A set of SQL queries were then executed to generate RDF triples using a set of PHP scripts. The scripts use SQL to query the database and output triples using print statements. Custom PHP scripts have been used as a lightweight solution to provide a Linked Data API via a Apache webserver, as well as to create data files with triples. These scripts are Open Source and are available from the source code hosting service, GitHub [11]. This process has been outlined in a best practices note by the W3C Health Care and Life Sciences Interest Group [12]. This process is similar to its original description.

However, the previous ChEMBL-RDF conversion used custom predicates and classes to represent RDF triples, defining an ad hoc ontology [8]. This implicit ontology reflects the concepts expressed in the relational database, and is not formally defined. The current version, instead, uses community-proposed ontologies, making the resulting RDF triples more interoperable with RDF provided by other life sciences databases. The new triple dataset uses standard ontologies including the Bibliography Ontology [13] and the Citation Typing Ontology (CiTO) [14] for literature references and citations, and domain ontologies such as the Protein Ontology[15] and the Chemical Information Ontology [16]. Throughout this paper various prefixes are used to denote different ontology namespaces to simplify the RDF examples. These prefixes are outlined in Table 1.

**Table 1:** Prefixes and their matching namespaces used in this paper

**Common vocabularies**	
bibo	Bibliography Ontology [13]
	http://purl.org/ontology/bibo/
chebi	Chemical Entities of Biological Interest [17]
	http://purl.org/obo/owl/CHEBI\#
cheminf	Chemical Information Ontology [16]
	http://semanticscience.org/resource/
cito	Citation Typing Ontology [14]
	http://purl.org/spar/cito/
obo / pro	OBO & PRotein Ontology [15]
	http://purl.obolibrary.org/obo/
bfo	Basic Formal Ontology [18]
	http://www.ifomis.org/bfo/1.1/snap\#
**ChEMBL-RDF Namespace**	
chembl	http://rdf.farmbio.uu.se/chembl/onto/\#
**ChEMBL-RDF Prefixes**	
act	http://linkedchemistry.info/chembl/activity/
assay	http://linkedchemistry.info/chembl/assay/
mol	http://linkedchemistry.info/chembl/molecule/
res	http://linkedchemistry.info/chembl/resource/

To expose the ChEMBL-RDF data two approaches have been adopted. First, a SPARQL endpoint is being hosted at Uppsala University, using the Open-Source Edition of the Virtuoso software [19]. Usage is free, but the allowed volume of querying is capped, based on the estimated computational effort. Second, resources have been made dereferencable via the http://linkedchemistry.info resource. The Kasabi platform [20] was used for a period of time, until that project was discontinued. The Uppsala SPARQL endpoint still uses resource URIs based on the Kasabi approach, following a similar URI pattern where linkedchemistry.info/chembl is replaced by data.kasabi.com/dataset/chembl-rdf.

Although many parts of the ChEMBL-RDF 13 dataset now use community based ontologies, some terms from the previous ad hoc ontology are still used when a community alternative is not yet available. These terms were created under the URI namespace http://rdf.farmbio.uu.se/chembl/onto/\#, and are referenced here using the prefix: *chembl*.

## Results

We present here the updated ChEMBL-RDF, including a description of the mapping process and the identification of links to other datasets. Because the primary purpose of this paper is to expose the ChEMBL data as linked data, less focus has been placed on ontologically capturing the fine details of the pharmacological literature. This is the reason why many concepts are not mapped to existing ontologies, though also the lack of ontologies with a clear open license, as defined by the Open Definition [21], restrict the options.

### Data Structure

For each of the common resource classes, a triple pattern was defined, following the data available in the relational database. Unless the ontological meaning of the data in the database was very clear, the classes are annotated with concepts as implicitly defined by the relational database. For example, the activity concept in the triples merely follows the activity table in the database. As such, the concept of chembl:Activity is implied by the content of that table in the relational database.

However, where the database content is well-defined, specific ontologies have been used. For example, where the database specifies literature from which data were extracted, the CiTO ontology is used. The choice of the ontologies is driven by the use cases and compatibility with other Linked Data projects, rather than formal ontological considerations. We have not observed ontological contradictions based on these choices.

**Figure 1:**
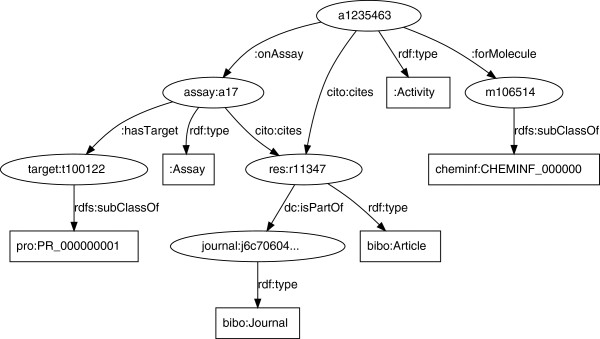
**The various resource types found in the ChEMBL-RDF triples.** Some entities are subclasses of common classes, using a rdfs:subClassOf predicate, such as target:t100122, while others are instances using the rdf:type predicate, such as assay:a17. The predicates between classes are also provided, showing how the resources are semantically linked.

Figure 1 shows how the various resource classes are linked together, detailed in this section at a triple level. The core concept in the ChEMBL database is that of the biological activity. While ChEMBL provides both the original activities as found in literature and standardized values allowing comparison between studies, the triples only make the latter available. The links to the literature are encoded using the CiTO ontology. This results in a set of triples that look like: 

More than five thousand different activity types, represented by the chembl:type predicate, are captured by the ChEMBL database. The top five types are “Potency” (43%), “IC50” (13%), “MIC” (4.6%), “Inhibition” (3.7%), and “Ki” (3.6%). The activity types in ChEMBL-RDF are currently not available as, or linked to, an ontology. The BioAssay Ontology could be a future option [22].

The activities themselves are measured against assays, which make up a second important resource type. Various assay types are found in the database: 

Each assay measures activities against one or more particular biological targets. To each target the ChEMBL database associates a confidence score (see the Compound Selectivity example). Because this score is specific for each target, the following construct is used: 

The ChEMBL database recognizes various target types: pro:PR_0000001, verbchembl:ADMET, chembl:CELL-LINE, chembl:NUCLEIC-ACID, chembl:ORGANISM, chembl:SUBCELLULAR, chembl:TISSUE, chembl:UNCHECKED, and chembl:UNKNOWN. The latter two are currently defined as explicit types, thus effectively implementing a closed-world approach.

A target specification can then look like: 

For drug discovery, the drug-like compounds themselves are the main topic of study. ChEMBL contains many different compound types, mostly small molecules, but also peptides, proteins, antibodies, oligosaccharides, oligonucleotides, and even cells. ChEMBL-RDF follows the approach used by the CHEMINF ontology, and encodes drugs as classes, rather than instances, and they subclass other classes defined by several ontologies for these types. For example, protein drugs subclass the Protein class from the PRotein Ontology (PR_000000001), instead that these drugs are of the Protein type. Each drug is a class itself. Likewise, small molecules subclass the Chemical Entity class from the CHEMINF ontology (CHEMINF_000000) (which is itself equivalent to the Chemical Entity class in ChEBI, CHEBI_24431), and oligosaccharides and oligonucleotides subclass their respective matches in the CHEBI ontology (CHEBI_50699 and CHEBI_7754). To ensure that we can look up all entities for which activities are reported, we subclass everything from the BFO 1.1 class MaterialEntity [18], which is a superclass of the CHEMINF Chemical Entity class.

It should also be noted that all drug-like entities in ChEMBL are not ontologically subclassing a drug class. Instead, we give the entities the role of drug, and only within a particular context would they be a drug. If not used as a medication, they are not a drug. Moreover, the triples only attach this drug role to approved drugs, as defined by the ChEMBL database. The role is triplified using the OBO and ChEBI ontologies in the following manner, where CHEBI_23888 is the ontological entry for “drug role” in ChEBI:

The name and synonyms for each drug are provided as labels. If SMILES, InChI, or InChIKeys are given for a drug, then these are provided via the CHEMINF formalism. The CHEMINF ontology has been used here because of its flexibility in defining descriptor types and their origin and meaning. By allowing an ontology of descriptors, we enable the comparison of values between databases semantically. Differences in, for example, the SMILES string may then be explained by the software used for that descriptor.

Example output for ChEMBL406142 looks like: 

Similarly, the properties of small molecules are available from ChEMBL, and as of ChEMBL-RDF 13 these too are exposed in triple format. Like the InChI and InChIKey, these are provided using the CHEMINF ontology. Here is, for example, the logP value (as distributed with ChEMBL and calculated with ACD/Labs software): 

The literature from which all this data was extracted, is replicated from the database, but only a subset of the possible information about that literature is provided. In all cases the PubMed Identifiers (PMIDs) and Digital Object Identifiers (DOIs) are used to link to an authoritative database, as outlined later in this paper. The properties are provided using the BIBO ontology, which is an open source ontology for bibliographic information, compatible with the CiTO ontology: 

### Data statistics and validation

Each release of the ChEMBL database is accompanied by a set of release statistics which help provide a concise content overview of the release in question. The ChEMBL 13 release counts are as follows: 

• 8,845 targets

• 1,143,682 distinct molecules

• 6,933,068 activities

• 617,681 assays

• 44,682 documents

These counts are important as firstly they show continued growth in ChEMBL data, as seen in all previous releases. Secondly, these counts can help validate any transformations, which have been applied to the relational data model traditionally used to store the ChEMBL data. The following SPARQL queries attempt to provide a high level validation of the ChEMBL-RDF by regenerating the ChEMBL 13 release statistics.

The following SPARQL query returns 8,845 ChEMBL targets: A similar SPARQL query, replacing chembl:Target with bfo:MaterialEntity, returns 1,143,682 entities for which activities are captured in ChEMBL, and by using chembl:Assay, the query returns the total of 617,676 ChEMBL assays. It should be noted that this last query returns five entries less than the SQL database, because the triple generation requires the assay type to be defined, which is not the case for those five assays. With chembl:Activity and bibo:Article, we count 6,933,068 ChEMBL activities and 44,681 documents, respectively. The document count is one fewer than appears in the ChEMBL 13 release notes [23], as the result does not include the doc_id -1, as this refers to the unpublished dataset, which is not a valid bibo:Article.

Reproduction of the ChEMBL release statistics through querying ChEMBL-RDF with SPARQL provides end users with a high degree of confidence that the ChEMBL-RDF contains the same core content as the source relational database. The development of a validation test suite is being discussed but not yet implemented; testing criteria other then the summarizing counts presented here, would change with each release due to curation events. Interactive, manual curation of the conversion process as employed by the authors already is more effective at this moment.

### Linked open data

**Figure 2:**
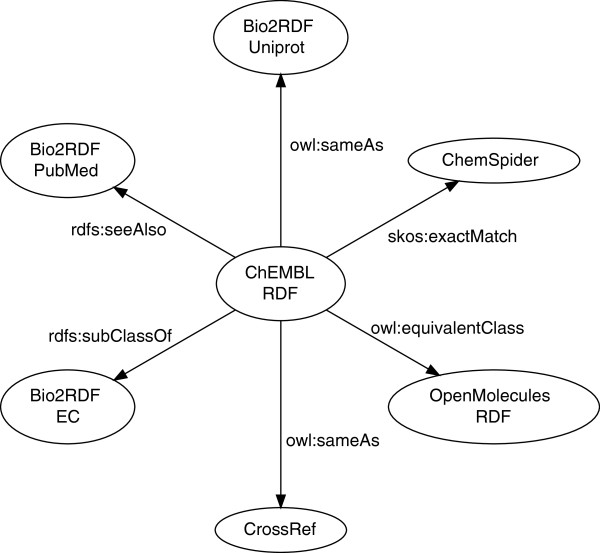
**The links out of the ChEMBL-RDF data into the Linked Open Data cloud versions of various external databases.** Edges are labeled by the predicates making the links.

To integrate ChEMBL-RDF with RDF versions of other scientific datasets, we link out to various resources. These links are shown in Figure 2. Triples for compounds link out to ChemSpider using the complementary index and OpenMolecules RDF using InChI values [8,24,25]. Protein links are given to Bio2RDF [2] using the UniProt identifier [26]. Literature references are directed to CrossRef and Bio2RDF using the DOIs and PubMed identifiers, respectively [27].

The predicates used to capture the links to these databases are rdfs:seeAlso, skos:exactMatch, owl:equivalentClass, and owl:sameAs. These relations are ordered according to their increasing level of implication: ranging from “you may also be interested in” with rdfs:seeAlso to “both objects are identical and all properties apply to both objects” for owl:sameAs.

#### Linking out to Bio2RDF

The Bio2RDF project provides both resolvable Linked Data URIs using a generic Linked Data server [28] and SPARQL endpoints containing RDF data from a range of scientific databases [2]. A number of these databases are referenced in ChEMBL, including ChEBI, PubMed, and both the UniProt protein and taxonomy databases [26]. These links are vital to provide context for use cases that require a correlation between chemical structures and other scientific data.

For resources from the biomedical with PubMed identifiers we also link out to Bio2RDF:

#### Linking out to ChemSpider

ChemSpider [24] is a freely accessible, online database provided by Royal Society of Chemistry (RSC). It contains over 28 million unique chemical compounds aggregated from over 400 data sources, as well as chemical data extracted from RSC scientific articles and databases. Since its inception, efforts have been made to utilize it as both a deposition platform for the community to contribute novel data, as well as a platform for annotation and curation for existing data. ChemSpider has become a valuable resource for curated data, especially chemical-compound name mappings [29]. ChemSpider is presently providing the chemical structure, substructure and similarity searching services underpinning the Open PHACTS semantic web project [4]. Specific chemical data sources containing data mappings between ChemSpider identifiers (CSIDs) and the original data source identifiers have been provided to the triple store, together with chemical identifiers including validated chemical names (systematic, generic and trivial), SMILES, and InChIs. The data mappings between the CSIDs and ChEMBL IDs are released to the community under the Creative Commons Attribution, Share Alike, license (CC-BY-SA 3.0). Attribution should be made to the original ChEMBL database, Open PHACTS, and ChemSpider.

Link mappings are provided with skos:exactMatch predicates, while the ChemSpider identifiers are also available via a CHEMINF representation:

#### Linking out to OpenMolecules RDF

The InChI is a unique identifier for (small) organic molecules, and has been previously used to define unique URIs for molecules [8,25]. While URIs are theoretically unlimited in length, in practice many web browsers and servers limit the length of URIs. Virtuoso is, unfortunately, one such system that supports only URIs of up to a certain length. Therefore, InChI-based links are only created for smaller molecules. Almost 1.1 million links to OpenMolecules RDF were created in a similar manner to:  Notice here the use of owl:equivalentClass since in CHEMINF molecules are defined as classes rather than instances.

#### Linking out to CrossRef

In addition to the PubMed identifiers used to link from literature references to Bio2RDF, ChEMBL provides DOIs, which we use to link out to the RDF provided by CrossRef [27]: 

res:r2032 owl:sameAs <http://dx.doi.org/10.1016/0960-894X(96)00111-4>.

## Applications

ChEMBL-RDF can be used to explore interrelated scientific datasets, and we present here five applications, describing how the SPARQL endpoint can be used, the combination of the ChEMBL data with other life sciences databases, the use of the bibliographic information in calculating citation statistics, and how to integrate ChEMBL-RDF with ChemSpider to provide an extension for the decision support platform in Bioclipse.

### Bio2RDF

This first application shows how ChEMBL-RDF can be integrated into other linked data services. The Linked Data server used by Bio2RDF has been reconfigured for ChEMBL to provide URL based services for standard URI resolution, along with textual and link-based searches [28]. A Java Web Archive (WAR) file along with the configuration files and build scripts for the ChEMBL-RDF Linked Data server are available on GitHub [30]. It proxies the standard ChEMBL-RDF URIs by translating URLs between those requested by users and the URIs that are available in SPARQL endpoints. For example, if the ChEMBL web application is running on the user’s local machine, e.g. http://localhost:8080/chembl/, then a request for the article with identifier “a31863”, http://localhost:8080/chembl/article/a31863, will be resolved using the webapp from the database using the full original URI, http://linkedchemistry.info/chembl/activity/a31863. If the user requested an RDF document using content negotiation, the original URIs will be unchanged, however, if the user requested an HTML document, the results will contain both the original RDF triples, represented using RDFa, with links that resolve using the user’s local machine.

These link services enable the ChEMBL application to derive both forward links, originating in ChEMBL, e.g. http://localhost:8080/chembl/linkstonamespace/-targetns/originalns:identifier, and backward links, originating in other databases, such as LODD, Bio2RDF (http://bio2rdf.org/linksns/targetns/originalns:identifier), and Chem2Bio2RDF. These services are vital to efficiently navigate the Linked Data web, as it is both impractical and inefficient to require users to crawl the entire web before they can discover relevant resources. These services are currently only supplied as web services from ChEMBL and Bio2RDF, but it is hoped that similar services will be provided by other scientific Linked Data providers in the future. Datasets that are available in SPARQL endpoints can be queried for links efficiently using simple queries as demonstrated in the ChEMBL web application.

### Chem2Bio2RDF

A second resource in the linked open data network into which ChEMBL-RDF was integrated is Chem2Bio2RDF. Chem2Bio2RDF is a single RDF repository covering over twenty public data resources pertaining to drugs, chemical compounds, carcinogens, protein targets, genes, diseases, side effects, pathways and their relations [7]. The entities and their relations were further annotated by Chem2Bio2OWL ontology, making it a rich semantic resource for integrative searches [31] and data mining [32]. The ChEMBL-RDF set was uploaded into the Chem2Bio2RDF triple store, enabling queries linking ChEMBL with other entities in Chem2Bio2RDF. Since both ChEMBL-RDF and Chem2Bio2RDF use InChI keys to present chemical compounds and adopt Bio2RDF protein identifiers to present targets, queries can be easily constructed to link the two. For instance, to investigate the relations between drug side effects and their targets (usually off-target effects), a query was created to link the targets in ChEMBL to side effects (e.g., heart disease) in Chem2Bio2RDF via side effect related drugs and their bioassay activities. In this case, 36 drugs causing heart disease were linked to 87 unique protein targets (*IC*_50_ < 10 *μ*m). The top two most common targets of these drugs are HERG and Serotonin transporter. HERG is a well-known off-target accounting for cardiovascular problems, while the activity of Serotonin transporter may also be associated with significant cardiovascular adverse effects [33]. The targets can also be linked to side effects via protein-protein interactions by searching across ChEMBL, HPRD and SIDER in Chem2Bio2RDF. Example SPARQL queries are available at http://chem2bio2rdf.wikispaces.com/Integrate+with+ChEMBL and can be executed in the SPARQL endpoint [7].

### CitedIn

**Figure 3:**
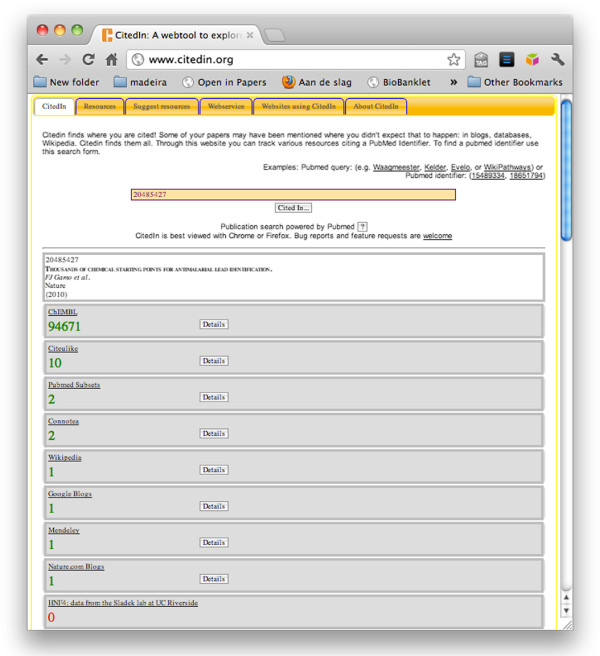
**Screenshot of the CitedIn web service showing a 2010 Nature paper cited 94671 times in the ChEMBL database.** The *Details* button on the webpage links to a ChEMBL webpage with detail on what parts of the database are linked to that paper.

Using the SPARQL endpoint, it is easy to integrate data from ChEMBL into other web resources, such as CitedIn (http://www.citedin.org) [34]. While the impact of a scientific publication is traditionally measured by frequency of citation in subsequent publications, this narrowly defined calculation increasingly fails to capture the true reach of a paper. CitedIn was developed to make scientific impact more visible and more accurate by providing insight into where published work is being cited online in a variety of public resources, as listed on the CitedIn website [35]. ChEMBL contains many literature references. The SPARQL endpoint is queried by CitedIn and the results are presented to its users. Figure 3 shows the results for the Nature paper by Gamo presenting the malaria structures [36] which is cited as the reference for a large number of activity datapoints and compounds in ChEMBL. Counting all objects that reference this paper is done by the following SPARQL query template: 

The CitedIn summary for ChEMBL also provides a *Details* button that links back to a ChEMBL webpage providing a *Document Report Card* showing in more detail what targets, assays, compounds, and activities in the database are linked to that paper.

### Bioclipse decision support

The use of the RDF open standard also simplifies the integration of data resources with scientific software, as we have shown previously [8,37]. We here demonstrate a new application following this idea.

Bioclipse Decision Support (Bioclipse DS) [38] is a user-oriented tool based on the Bioclipse workbench for providing on-time and on-demand information on chemical structures. Such information can include calculated properties, data from database queries, and results from predictive models. Bioclipse DS has previously been demonstrated on predictive modeling in drug safety assessment [38] and also been linked to invoke and present results from distributed toxicity predictions from the OpenTox infrastructure [37].

**Figure 4:**
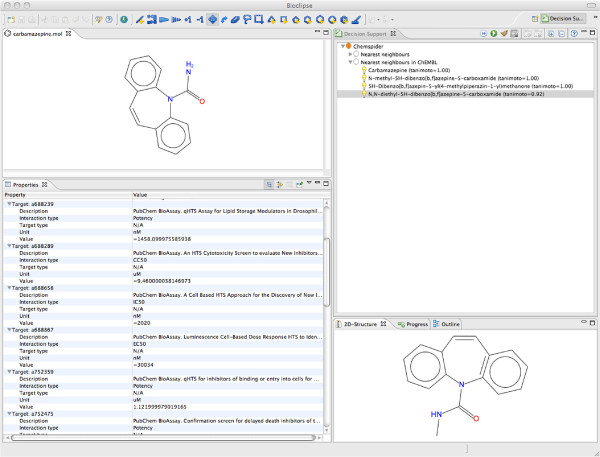
**Screenshot from the Bioclipse Decision Support with results from a combined ChemSpider and ChEMBL-RDF search.** The top left canvas contains the query structure, carbamezapine, and the top right canvas shows its near neighbors found in ChemSpider for which ChEMBL-RDF data exists. The lower right canvas shows the chemical structure selected in the top right canvas, and the lower left canvas shows the available activities in ChEMBL-RDF for this compound.

In this study we extended Bioclipse DS with remote access to both ChEMBL-RDF and ChemSpider. This enables users browsing chemical structures in Bioclipse to look up nearest neighbors in ChemSpider via the ChemSpider Web API (SOAP), and for the found compounds to be used to query ChEMBL-RDF for available interaction data. The results are presented alongside predictive models in Bioclipse (see Figure 4), and can be used for decision support when evaluating chemical structures and considering strategies for optimization.

### Compound selectivity

Designing a molecule that is selective in binding to one target rather than another is often considered a successful outcome in a drug design process. If the target in question is a protein, high sequence similarity between the binding site and that of other proteins will make such selectivity more challenging. That is, the molecule being designed may also bind to the equivalent binding site in the closely related protein target, and this may lead to undesirable consequences. The ChEMBL data model links molecules to targets using different activity types recorded in the literature. Using activity types that act as a measure of binding affinity, such as *IC*_50_ or *K*_*i*_, and applying activity value cut-offs, it is possible to identify molecules which have higher binding affinity to certain targets compared to others. Taking this one step further, it is possible to identify a set of molecules that have a high affinity to protein A (e.g. *IC*_50_ value < 50 nM) and low affinity to protein B (e.g. *IC*_50_ value > 200 nM).

The following SPARQL query identifies a set of 65 molecules that selectively bind Human Cyclin-Dependent Kinase 2 (UniProt: P24941) over Human Cyclin-Dependent Kinase 4 (UniProt: P11802), based on data curated from the literature and stored in the ChEMBL-RDF data model.

Analysis on the set of molecules returned by this query can be used to help identify small molecule features, which may increase target-binding specificity. For queries which link small molecules to targets, by traversing bioactivity data in the ChEMBL database, it is also important to consider the parameters associated with the assay to target mappings. These additional parameters include a relationship type, a multi flag (for poorly defined targets) a complex flag (for protein complex targets) and a curation level. These different factors are summarized in the ChEMBL confidence score, which ranges from 9 (direct single protein target) to 0 (uncurated). In order to return the largest possible dataset, the confidence score has been ignored in this example compound selectivity use case.

## Discussion

This paper shows the evolution of the ChEMBL-RDF resource in standardizing access to the ChEMBL data in a machine-readable manner using semantic web technologies. We showed here that the conversion into RDF allows a number of novel integrations with other resources. It should be clear that these applications can be made using other technologies too, but using the RDF standards it is easier to implement them. More importantly, the solutions will be generally applicable to any system using these open standards. However, a number of choices need discussion.

Much could be argued about the choice of ontologies, and the lack of an ontology in places where we decided to use custom classes and predicates. Most of the choices are based on pragmatic approaches to enable solutions to the range of use cases we describe here. It should be noted, however, that the ChEMBL database captures a wide variety of experimental studies and that semantics are not fully specified in the primary source from which the ChEMBL data was extracted. As it is not possible to infer the original intentions in these cases, it is our opinion that it is better to leave some semantics undefined. During the development of the applications some incorrect assumptions have been discovered and fixed, but in the main we have tried to avoid making assumptions if they are not generally known to be valid. Therefore, the goal of this work is the conversion of the ChEMBL data into RDF, and not an ontological modeling of the domain.

One case where Linked Data publishers must avoid implying strong semantics in many cases is in relation to links to other databases. When linking between two databases based on a shared identifier, Linked Data publishers make assumptions including syntax assumptions that the identifiers are correct and present in the target database, along with semantic assumptions inferring that the that entries identified in the target database have a scientifically meaningful relationship with the current database. It is for this reason that the ChEMBL-RDF dataset uses different predicates for linking to various databases. This problem, however, is general, applying also to other cases where databases link out to third-party sources using identifiers [39].

Also debatable is the choice of ontologies used to describe the dataset in RDF. As an example, in this release of the ChEMBL-RDF data, we adopted the cheminformatics ontology, CHEMINF. Using this ontology we can encode numeric and non-numeric descriptors of chemical entities in a consistent manner. This method is used to consistently expose identity descriptors such as the SMILES string, InChI, and InChIKey, and molecular properties including the logP, rotatable bond count, and hydrogen bond donor and acceptor counts. However, the design of this annotation is more general than may be needed for a translation of the data in the ChEMBL relational database into RDF. Although this results in more complex SPARQL queries, it allows generic queries that may not otherwise be easy to construct. While the selection of ontologies used was found to fit well with our applications, we anticipate that we will select more expressive ontologies in future iterations of the project.

Some information in the databases has not yet been annotated using standard ontologies. For example, the activity types in ChEMBL, such as “IC50”, “EC50”, and “Potency”, are not exposed in the RDF dataset, meaning that it is not yet possible to reason or group over similar activity types. When the activity types are exposed, queries could be constructed based on the knowledge that the IC_50_ and K _*i*_ endpoint are closely related. Similarly, measurement units are currently provided as string literals, making it difficult to compare concentrations when some are given in nM and others in mM. In the future it may also be possible, relying on some assumptions, to crossmatch activities expressed in *μ*g/ml with query concentrations in nM. These additional semantics are necessary to translate raw experimental data into semantically useful, machine-readable information.

Two ontologies were recently developed that may address these issues for ChEMBL-RDF in future. Firstly, the BioAssay Ontology, which addresses many of our remaining concerns around making the experimental data machine readable. For example, it provides an ontology of activity types. Secondly, the Quantity, Unit, Dimension and Type (QUDT) ontology which makes it possible to express measurements in a standardised machine readable way [40]. Both approaches are being explored in the Open PHACTS project and will be applied to ChEMBL-RDF as the Open PHACTS project progresses [4].

## Conclusions

Taking these points into account, we believe that the adoption of RDF and community based ontologies allows novel use cases to be addressed using the ChEMBL data that would not otherwise be achievable. More importantly, the adoption of explicit semantics simplifies integration with other resources, as others can more easily understand the intent and purpose of each part of the ChEMBL dataset. This is a requirement which is essential to the efforts of cheminformatics researchers wishing to infer knowledge from ChEMBL as well as other datasets.

The ongoing ChEMBL-RDF project is Open Source making it possible to others to convert ChEMBL to RDF too. Technically, the choice of PHP makes it easy to share functionality between the here described conversion scripts, and PHP-enabled web services, used to provide the Linked Data interface to the ChEMBL-RDF data, as is fast enough for practical uses. These scripts do not use a library for handling RDF data. While using such libraries increases the dependencies for the scripts, they also ensure the generation of syntactically correct RDF triples. Continued development of the ChEMBL-RDF scripts, however, has started using RDF libraries for this purpose specifically for triple generation of compound names, removing code in the scripts to escape special characters, like double quotes.

Finally, the ChEMBL project will provide RDF versions of ChEMBL, while others continue exposing ChEMBL as RDF too (EW, PA). In the future various versions may arise, exposing the data in different ontologies, supporting current community needs, however, convergence is expected after a period of experimenting with ontologies that may not prove to be practically useful. The availability of Open Source scripts ensures that the community can contribute to the process of choosing a practically useful set of ontological terms.

## Competing interests

The authors declare that they have no competing interests.

## Authors’ contributions

EW initiated the project, created the initial RDF version of the ChEMBL data, and encouraged the use cases. AW extended CitedIn to support citation info in ChEMBL-RDF and supported setting up the http://linkedchemistry.info/ host. OS, AJW, and VT developed the nearest neighbor application of Bioclipse and ChemSpider. JH supported the project with CHEMINF representations. PA integrated ChEMBL-RDF into Bio2RDF. BC, DW contributed the Chem2Bio2RDF use cases. All authors contributed to the continued development of ChEMBL-RDF, as well as to the writing of the paper, and approved the final version. All authors read and approved the final manuscript.
